# Seasonal Trends and Site Differences of Nitroaromatic Compounds in PM_2.5_ in Sichuan Basin and Their Effects on Light Absorption of Brown Carbon

**DOI:** 10.3390/toxics13020124

**Published:** 2025-02-06

**Authors:** Tian Tang, Buyi Xu, Hongli Tao, Tingting Huo, Huanbo Wang

**Affiliations:** 1 School of Environment and Resource, Southwest University of Science and Technology, Mianyang 621010, China; tangtian_fu99@163.com (T.T.); hltao_11@mails.swust.edu.cn (H.T.); huotingting1203@163.com (T.H.); 2 Sichuan Meteorological Disaster Prevention Technology Centre, Chengdu 610072, China; 3 Sichuan Provincial Department of Public Security, Chengdu 610041, China; xubuyi123@scpolicec.edu.cn

**Keywords:** nitrocatechols, brown carbon, optical properties, spatiotemporal variation, Chengdu

## Abstract

Nitroaromatic compounds (NACs) have adverse effects on human health and climate. Daily PM_2.5_ samples were collected in winter and summer of 2022 in two cities, Chengdu (CD) and Mianyang (MY), located in Sichuan Basin of southwestern China. Four types of NACs in PM_2.5_, containing nitrophenols, nitrocatechols, nitrosalicylic acids, and nitronaphthol, were analyzed. The mean concentration of a total of 10 NACs (ΣNACs) in winter at the suburban MY site (71.7 ± 35.6 ng m^−3^) was higher than that in urban CD (29.5 ± 16.2 ng m^−3^), while in summer, the mean concentrations of ΣNACs in the two cities were similar, around 2.2 ng m^−3^. The much higher concentrations of ΣNACs in winter were attributed to the impact of biomass burning. 4-Nitrocatechol (4NC) was the most abundant species during the sampling period, accounting for 35–56% of ΣNACs mass. In winter, the mean light absorption coefficient of methanol-soluble brown carbon (Abs_365,M_) was 10.5 ± 3.4 and 13.6 ± 4.3 Mm^−1^ in CD and MY, respectively, which was about 4–7 times that of summer. The contributions of light absorption of ΣNACs at 365 nm to Abs_365,M_ were 1.6–3.6% in winter and 0.5–0.7% in summer, with 4NC contributing the most to brown carbon among all NACs. The geographical origins of potential sources of NACs at both sites were mainly distributed within the basin.

## 1. Introduction

Carbonaceous aerosols are of increasing concern with regard to their impact on global climate change, air quality, and human health [1,2,3,4]. A fraction of organic carbon (OC) that can absorb radiation in the near-ultraviolet and visible ranges is referred to as brown carbon (BrC) [5,6]. The molecular composition of BrC is complex, and only a few chromophores have been identified. Budisulistiorini et al. [7] identified 41 light-absorbing substances using an ultra-performance liquid chromatography instrument interfaced with a diode array detector and electrospray ionization high-resolution quadrupole time-of-flight mass spectrometer (HPLC-DAD-Q-TOF MS), showing that BrC constituents accounted for only 0.4% of the ambient organic aerosol mass. Huang et al. [8] identified 18 chromophores in water-soluble BrC and 16 chromophores in water-insoluble BrC using a high-performance liquid chromatograph equipped with a photodiode array and a high-resolution Orbitrap mass spectrometer (HPLC-PDA-HRMS). Results showed that nitrophenols, polycyclic aromatic hydrocarbons, and carbonyl oxygenated polycyclic aromatic hydrocarbons were dominant chromophores of BrC, which explained 6–14% of the light absorption of BrC. Among these chromophores, nitroaromatic compounds (NACs) were identified as important light-absorbing compounds of BrC [8,9]. For example, Desyaterik et al. [10] determined BrC in cloud water impacted by agricultural biomass burning in eastern China, indicating that nitrophenol derivatives and aromatic carbonyls were the most important light-absorbing species. Lin et al. [11] investigated the chemical composition of BrC using HPLC-PDA-HRMS, demonstrating that nitroaromatic compounds were the primary chromophores of BrC during a biomass burning event. Zhang et al. [9] also showed that the main BrC chromophores in Shanghai were NACs (i.e., nitrophenol and nitroaromatic acid) through HPLC-DAD-Q-TOF MS.

There are significant differences in the spatial distribution and seasonal trends of atmospheric NAC concentrations and compositions. In 2017, the annual mean concentration of total NACs in Nanjing was 26.48 ng m^−3^, with higher concentrations in winter and lower in summer. Moreover, nitrophenols and nitrocatechols were identified as the dominant components of NACs in winter, while nitrosalicylic acids accounted for about 85% in summer [12]. Daily concentrations of total NACs in Xi’an were in the range of 0.1–127 ng m^−3^, with mean concentrations of 1.1–56 ng m^−3^ in four seasons. Among all the measured NACs, 4-nitrophenol (4NP) and 4-nitrocatechol (4NC) showed higher concentrations than the other NACs in all seasons [13]. The seasonal mean concentration of nine NACs in Beijing was similar in spring (8.58 ng m^−3^) and summer (8.54 ng m^−3^), and the most abundant components in spring and summer were 4NP and 4-nitroguaiacol, respectively [14]. A comprehensive sampling campaign was carried out in urban, rural, and mountainous areas in northern China during the summer. Results showed that the mean concentration of total NACs in urban Jinan was the highest (9.8 ng m^−3^), followed by the rural areas of Yucheng (5.7 ng m^−3^) and Wangdu (5.9 ng m^−3^), while the concentration in the mountainous area of Mt. Tai was the lowest (2.5 ng m^−3^) [15]. The concentrations of NACs in the mountainous background of Mt. Wuyi also showed relatively lower levels, with seasonal mean values of 1.3 ± 0.75, 0.97 ± 0.36, 2.1 ± 0.94, and 3.9 ± 1.5 ng m^−3^ in spring, summer, autumn, and winter, respectively [16].

Although NACs only accounted for a small fraction of the OC mass, their contribution to BrC light absorption cannot be ignored. For example, at a rural site in the eastern Indo-Gangetic Plain, atmospheric NACs accounted for only 0.42 ± 0.23% of the OC mass. However, the contribution of NACs to BrC light absorption in the range of 300–450 nm was more than an order of magnitude higher (8 ± 4%) [17]. Frka et al. [18] also found that the contribution of NACs to the light absorption of the aqueous extracts was 10 times higher than the mass concentration of water-soluble OC. Li et al. [19] showed that NACs accounted for 1.17% and 3.18% of the light absorption of methanol-soluble BrC in Beijing in autumn and winter, respectively, while NACs contributed only 0.16% and 0.44% of the OC mass, suggesting that NACs were strong light-absorbing substances in BrC. Zhang et al. [9] identified nine NACs in ambient aerosols in Shanghai, of which 4NP was found to be the strongest light-absorbing substance, accounting for 13% of the total light absorption. Yuan et al. [13] found that the contribution of atmospheric NACs to the light absorption of BrC in urban Xi’an was wavelength-dependent and varied largely with the season. e.g., the average contributions of NACs to the light absorption of BrC were 0.14%, 0.09%, 0.36%, and 0.91% in spring, summer, autumn, and winter, respectively, which were about 6–9 times their mass fractions in total organic carbon.

As mentioned above, the contributions of NACs to light absorption of BrC varied considerably from city to city, which were probably attributed to the different proportions of individual NACs due to different major sources and meteorological conditions in different areas. So far, research on the characteristics of atmospheric NACs and the role of NACs in BrC light absorption has mainly focused on northern [20,21,22], eastern [9,12,23], and northwestern China [24], with few studies being conducted in Sichuan Basin [25], especially on the light absorption properties of NACs. Considering intensive biomass burning events and special meteorological conditions such as high relative humidity and low wind speed all year round in Sichuan Basin [26], investigating the role of NACs on BrC light absorption in this region is necessary and crucial to understanding the impact of NACs on climate change in the basin.

In this study, PM_2.5_ samples were collected in Chengdu and Mianyang in the winter and summer of 2022. The main aims were (1) to investigate the seasonal trends and site differences of 10 NACs in urban and rural areas of the Sichuan Basin, (2) to evaluate the contribution of NACs to the light absorption of BrC, and (3) to identify the potential geographic regions of NACs during the sampling period.

## 2. Materials and Methods

### 2.1. PM_2.5_ Sampling

**Figure 1:**
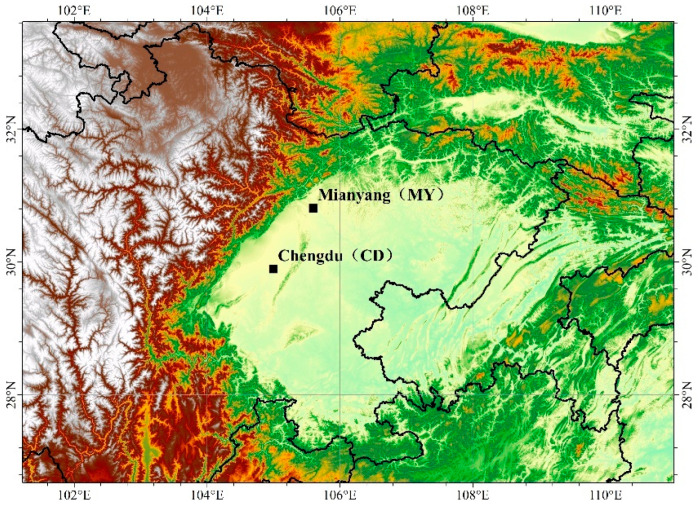
Locations of sampling sites in CD and MY.

PM_2.5_ samples were collected in two cities in the Sichuan Basin, one in Chengdu and the other in Mianyang. The sampling site in Chengdu (CD) is located at the Wangjiang Campus of Sichuan University (30.63° N, 104.09° E), an urban site that is likely impacted by local vehicular, residential, and commercial emissions [27]. The sampling site in Mianyang (MY) is situated on the campus of Southwest University of Science and Technology (31.54° N, 104.69° E), surrounded by restaurants and commercial areas, with no industrial pollution sources nearby [28]. The locations of the two sampling sites are shown in Figure 1.

PM_2.5_ samples were collected on quartz filters (8 × 10 inches, Whatman or PALL) using a high-volume sampler (TE-6070, Tisch Inc., USA). The quartz filters were preheated at 450 °C for 6 h before use in order to remove organic compounds. 23-h integrated PM_2.5_ samples were collected in CD and MY over two seasons as follows: in winter in CD (1–21 January 2022) and in MY (19 December 2021 to 13 January 2022), in summer in CD and MY (June–August 2022). During the sampling period, PM_2.5_ samples were collected continuously in winter and approximately every 6 days in summer. In all, a total of 21 and 25 samples were collected in winter in CD and MY, respectively, while a total of 14 samples were collected in both cities in summer. Field blank filters were also collected in each season.

### 2.2. Chemical Analysis

Ten NACs, including 4-nitrophenol (4NP), 2-methyl-4-nitrophenol (2M4NP), 3-methyl-4-nitrophenol (3M4NP), 2,4-dinitrophenol (2,4DNP), 2,6-dimethyl-4-nitrophenol (2,6DM4NP), 4-nitrocatechol (4NC), 4-methyl-5-nitrocatechol (4M5NC), 3-nitrosalicylic acid (3NSA), 5-nitrosalicylic acid (5NSA), and 4-nitro-1-naphthol (4N-NaP), as well as isotopically labeled internal standard 4-nitrophenol-2,3,5,6-d_4_ (4NP-d_4_), were provided by Sigma-Aldrich (USA), Toronto Research Chemicals (Canada), J&K Scientific (China), and AccuStandard Inc. (USA).

A portion of quartz filter with an area of 8.67 cm^2^ was used in CD in winter and summer, while a piece of quartz filter with an area of 8.31 cm^2^ (winter) and 16.62 cm^2^ (summer) was punched in MY. Then, 100 μL of 2 μg mL^−1^ 4NP-d_4_ was spiked into the quartz filter. The punched quartz filter was extracted ultrasonically in methanol for 20 min, and the extract was then filtered through a 0.45 μm PTFE syringe filter to remove insoluble particles. The final extract was diluted to 10 mL with methanol.

Ten NACs were quantified using liquid chromatography-mass spectrometry/mass spectrometry (LC-MS/MS, SCIEX Triple Quad^TM^ 6500+, AB SCIEX, USA) equipped with an electrospray ionization source (ESI) in negative ion mode. The separation of the 10 NACs was performed on an ACQUITY UPLC BEH C18 column (2.1 mm × 100 mm, 1.7 μm, Waters, USA). The injection volume was 3 μL, and the column temperature was 40 °C. The mobile phase consisted of Milli-Q water with 0.1% formic acid (LC-MS grade, eluent A) and acetonitrile (HPLC grade, eluent B). The gradient elution protocol was adopted by Tao et al. [25] as follows: eluent B was initially kept at 5%, 0–1.8 min 5–20% B, 1.8–4.0 min 20% B, 4.0–11.0 min 20–60% B, 11.0–11.1 min 60–100% B, 11.1–13.0 min 100% B, 13.0–13.1 min 100–5% B, 13.1–15.0 min 5% B. The mass spectrometric analysis was performed in multiple reaction monitoring (MRM) mode. The precursor and product ions of the 10 NACs, mass spectrometer parameters, and the quantitative method performance of the target NACs can be found in our previous study [25].

Water-soluble inorganic ions (such as K^+^ and Cl^−^) were determined by ion chromatography (ICS-6000, Thermo Fisher, USA), as detailed in our previous study [28]. Briefly, a portion of a quartz filter (8.67 cm^2^) was punched in CD during the winter and summer sampling periods, and a piece of quartz filter of 8.31 cm^2^ and 16.62 cm^2^ was used in MY in winter and summer, respectively. Punched quartz filters were extracted ultrasonically with 10 mL Milli-Q water, and then extracts were filtered through a 0.45 μm syringe filter to remove insoluble particles. Cations (i.e., K^+^) were separated through a CS12A column with 20 mM MSA, and anions (i.e., Cl^−^) were determined by an AS11-HC column with 30 mM KOH.

Daily NO_2_ and PM_2.5_ concentrations were obtained from nearby ambient monitoring stations that were located approximately 2 km from the sampling site of CD (30.63° N, 104.07° E) and 1 km from the MY site (31.53° N, 104.69° E), respectively.

### 2.3. Light Absorption Analysis

The light absorption spectra of the methanol extracts were measured using a UV-Vis spectrophotometer (U-2900, Hitachi, Japan) in the wavelength range of 220–900 nm, with a scanning speed of 400 nm min^−1^ and a wavelength increment of 1 nm. Methanol was used for baseline correction before each analysis.

The light absorption coefficient of BrC at a certain wavelength of λ (Abs_λ_, Mm^−1^) was calculated as follows:

(1) $${\mathrm{Abs}}_{\lambda }=(A_{\lambda }-A_{700})\;\times \;\frac{V_{l}}{V_{a}\times L}\times \ln\left(10\right)$$

where A_λ_ and A_700_ are the light absorbance of the methanol extract at a certain wavelength of λ and 700 nm, respectively. V_l_ is the volume of the extract (10 mL), V_a_ is the volume of sampled air (m^3^), and L is the optical path length (0.01 m).

The light absorption coefficient of NACs at 365 nm (Abs_NACs,365_) was estimated according to the measured individual NAC concentrations (C_NACs_, ng m^−3^) and the mass absorption efficiency of individual NACs (MAE_NACs,365_, m^2^ g^−1^ C) as follows:

(2) $${\mathrm{Abs}}_{\mathrm{NACs},365}={\;\mathrm{MAE}}_{\mathrm{NACs},365}\;\times {\;C}_{\mathrm{NACs}}$$

### 2.4. Air Mass Backward Trajectories

To identify the potential source regions of NACs, 48-h air mass backward trajectories were performed using a hybrid single particle Lagrangian integrated trajectory model (HYSPLIT). The altitude of the air mass reaching the sampling site in winter and summer was set to 100 m and 300 m, respectively. During the sampling period, air trajectories were calculated every 6 h (UTC 02:00, 08:00, 14:00, 20:00), and clustering analysis was performed using the MeteoInfo version 3.8 software based on the Euclidean distance (http://meteothink.org/index.html, accessed on 6 January 2025).

## 3. Results and Discussion

### 3.1. Seasonal Variations in NACs at Urban and Suburban Sites

Table 1 summarizes the concentrations of NACs in PM_2.5_ during the winter and summer periods in CD and MY. In CD, daily concentrations of the 10 NACs (ΣNACs) ranged from 7.7 to 77.0 ng m^−3^ and 0.7 to 3.7 ng m^−3^ in winter and summer, respectively, with seasonal mean values of 29.5 ± 16.2 and 2.2 ± 1.1 ng m^−3^, while the corresponding concentrations in MY varied from 19.7 to 159.7 ng m^−3^ and 0.8 to 4.9 ng m^−3^, with mean values of 71.7 ± 35.6 and 2.5 ± 1.2 ng m^−3^, respectively. In summer, compared with the values in other Chinese cities, the seasonal mean concentrations of ΣNACs in CD and MY were at a lower level, e.g., lower than those in Guangzhou [24], Harbin [24], Beijing [14], Shanghai [29], and Nanjing [12], but higher than that in Xi’an [13] and the Mt. Wuyi background site [16]. Although Mt. Tai is a background site, the concentration of ΣNACs in Mt. Tai in summer was higher than that in CD and MY, possibly influenced by neighboring anthropogenic activities [30]. Compared to the winter NAC levels, the concentrations of ΣNACs in CD and MY were lower than those in Harbin [24], Beijing [22,31,32], and Qingdao [20,33], as these cities are located in northern and northeastern China, where increased heating demand in winter resulted in increased primary source emissions (e.g., biomass burning and coal combustion). In winter, the seasonal mean concentration of ΣNACs in CD was lower than those in Wuhan [24], Xi’an [13], Shanghai [29], and Nanjing [12,23], but the mean concentration of ΣNACs in MY was higher than that in the above cities, suggesting that the NACs pollution in Sichuan Basin should be given more attention, particularly in suburban areas.

**Figure 2:**
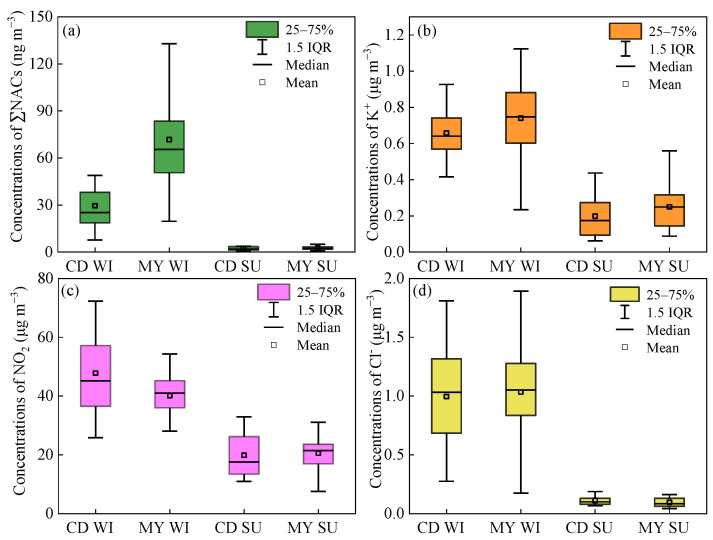
Seasonal variations in mass concentrations of ΣNACs (**a**), K^+^ (**b**), NO_2_ (**c**), and Cl^−^ (**d**) in winter (WI) and summer (SU) in CD and MY.

In terms of seasonal trends (Figure 2), the seasonal mean concentrations of ΣNACs in winter were about 13 and 30 times those in summer in CD and MY, respectively. The significant difference in ΣNAC concentrations between winter and summer was related to meteorological conditions and the sources and formation processes of NACs. In general, wind speeds and the boundary layer in the Sichuan Basin are lower in winter, which is unfavorable to the diffusion of pollutants; moreover, rainfall is not abundant in winter, which is not beneficial to the removal of pollutants from the atmosphere, resulting in the accumulation of pollutants in winter. In addition, lower temperatures in winter favor the transfer of semi-volatile NACs to the particulate phase through gas–particle partitioning, leading to more NACs being present in the particulate phase. On the contrary, higher temperatures in summer may result in more NACs being present in the gas phase. Moreover, the higher boundary layer and abundant rainfall in summer are conducive to the diffusion and removal of pollutants in the atmosphere [26]. Biomass burning and secondary formation are the main sources of NACs. K^+^ can be used as a tracer for biomass burning. As shown in Figure 2, the concentrations of K^+^ in CD and MY in winter were 3.3 and 3.0 times those in summer, respectively, indicating that biomass burning played an important role in the high concentrations of NACs in winter. In addition to primary emissions of NACs, biomass burning also emits a large amount of phenolic compounds (e.g., phenol, cresol), which promote the secondary formation of NACs at higher NO*x* concentrations. The concentrations of NO_2_ in CD and MY in winter were 2.4 and 1.9 times higher than those in summer, respectively (Figure 2), leading to an increase in the secondary formation of NACs. Regarding site differences, the seasonal mean values of ΣNACs in CD and MY in summer were comparable. However, the mean concentration of ΣNACs in MY in winter was about 2.4 times higher than that in CD, suggesting that there might be specific emission sources or special meteorological conditions in MY in winter that were more conducive to the formation of NACs, resulting in the concentrations of ΣNACs at the suburban site being much higher than that at the urban site.

**Figure 3:**
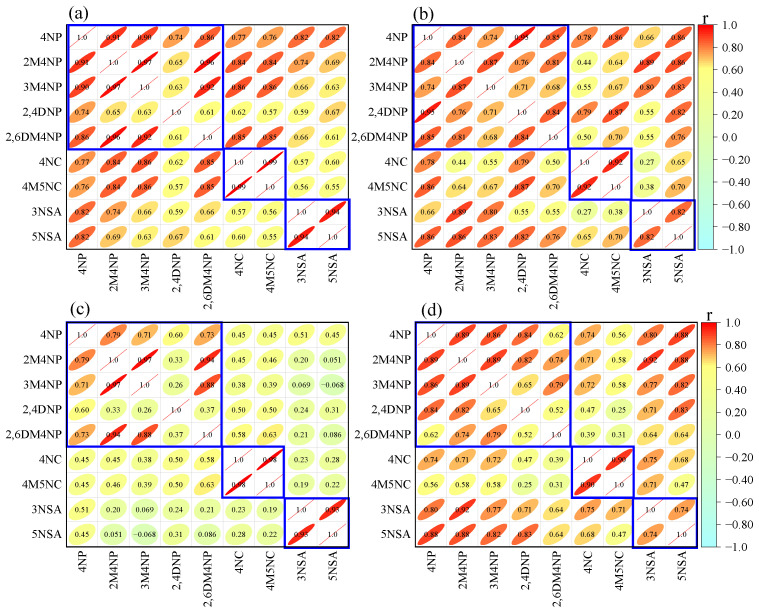
Correlations between nine NACs in winter (**a**,**c**) and summer (**b**,**d**) in CD (**upper panel**) and MY (**lower panel**), respectively.

**Figure 4:**
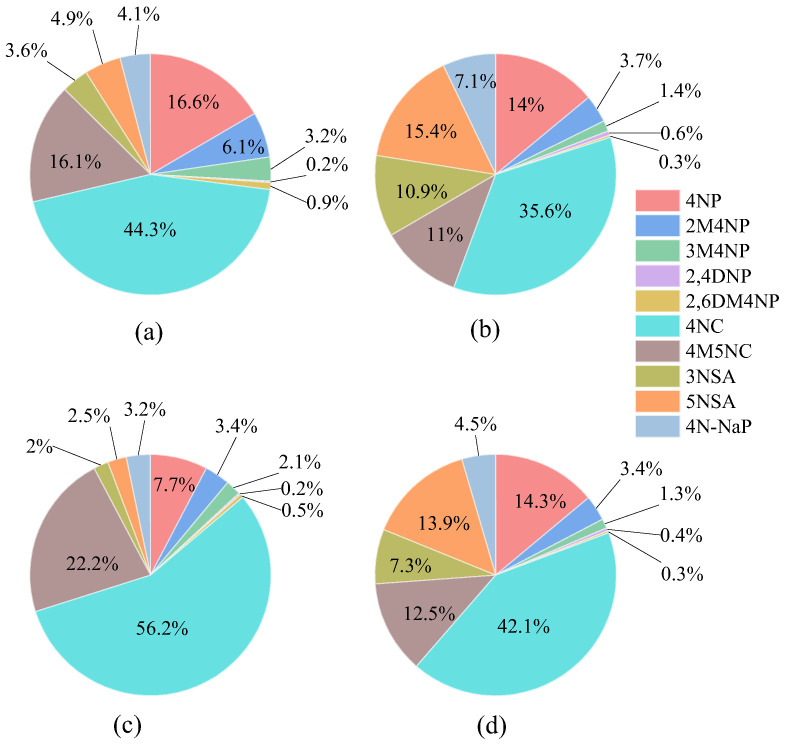
Contributions of 10 NACs to ΣNACs in winter (**a**,**c**) and summer (**b**,**d**) in CD (**upper panel**) and MY (**lower panel**), respectively.

As shown in Figure 3, there are good correlations between NAC compounds with similar structures. For example, strong correlations between 4NC and 4M5NC were observed in both winter and summer at CD and MY, with correlation coefficients (r) higher than 0.9. The same was true for the relationship between 3NSA and 5NSA, with the values of r in the range of 0.7–0.9. Except for 2,4DNP, the four nitrophenols (4NP, 2M4NP, 3M4NP, and 2,6DM4NP) also showed good correlations, with correlation coefficients above 0.7. For simplicity, the 10 NACs were divided into four groups according to their structures: NPs (4NP, 2M4NP, 3M4NP, 2,4DNP, 2,6DM4NP), NCs (4NC, 4M5NC), NSAs (3NSA, 5NSA), and 4N-NaP. As shown in Figure 4, NCs were the most abundant species among the four NACs groups. In CD, daily contributions of NCs to ΣNACs ranged from 47.1% to 76.7% in winter, with an average value of 60.4%, while daily percentages of NCs in ΣNACs in summer varied from 30.5% to 67.4%, with an average value of 46.6%, which was lower than that in winter. In MY, daily contributions of NCs to ΣNACs in winter were in the range of 55.8–88.3%, with an average value of 78.4%, which was much higher than that in CD, indicating that specific emission sources in winter had a greater impact on NCs in MY. Daily percentages of NCs in ΣNACs in summer ranged from 37.8% to 68.2%, with an average value of 54.6%, which was slightly higher than that in CD. Of the four NAC categories, 4N-NaP was the least abundant. The average percentage of 4N-NaP in ΣNACs in CD was 4.1% and 7.1% in winter and summer, respectively, whereas 4N-NaP in MY was less than 5% in both seasons.

The contributions of NPs and NSAs to ΣNACs were related to the seasons. The fractions of NPs in winter were much higher than those of NSAs. For example, in CD, daily contributions of NPs and NSAs to ΣNACs in winter were 16.5–36.9% and 3.6–14.7%, with average values of 27.0% and 8.5%, respectively. In MY, daily percentages of NPs and NSAs in ΣNACs in winter varied from 6.3% to 33.0% and 1.7% to 11.0%, with mean values of 13.9% and 4.5%, respectively. On the contrary, the amounts of NPs in summer were slightly lower than those of NSAs. For example, the average percentages of NPs and NSAs in ΣNACs in CD in summer were 20.1% and 26.3%, respectively, while the corresponding percentages were 19.3% and 21.6% in MY, respectively. The decrease in the contribution of NPs in summer may be strongly influenced by temperature, as NPs mainly exist in the gas phase at higher temperatures. Rana et al. [17] found that 4NP mainly existed in the gas phase, with a percentage in the particulate phase of less than 5%, whereas 3NSA and 5NSA mainly existed in the particulate phase. Huang et al. [24] found that the concentrations of NPs (e.g., 4NP, 2M4NP, 3M4NP) in Harbin, Chengdu, and Xi’an in the gas phase were much higher than those in the particulate phase, but NSAs were distributed entirely in the particulate phase. Li et al. [34] also showed that 70% of the NPs existed in the gas phase, while all the NSAs were present in the particulate phase in summer. Therefore, NPs were greatly affected by temperature. High temperatures promoted the volatilization of NPs into the gas phase, resulting in a lower concentration of NPs in the particulate phase in summer.

**Figure 5:**
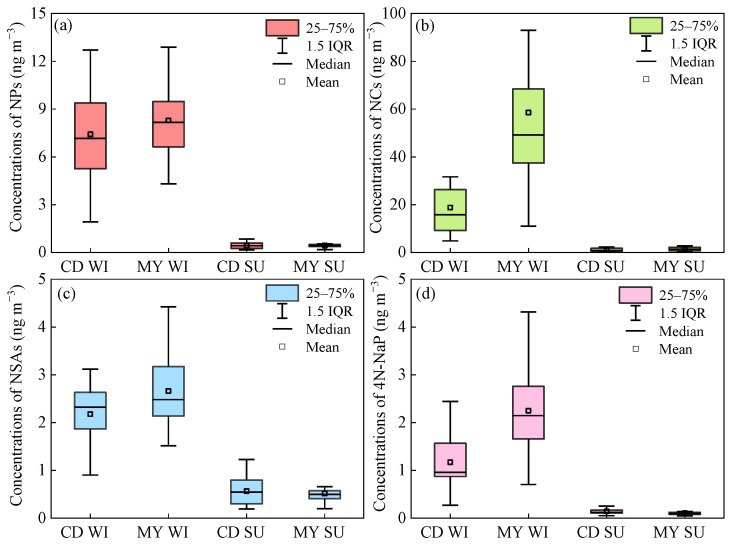
Seasonal variations of mass concentrations of NPs (**a**), NCs (**b**), NSAs (**c**), and 4N-NaP (**d**) in winter (WI) and summer (SU) in CD and MY.

As shown in Figure 5, the seasonal trend of NCs is consistent with ΣNACs. The concentrations of NCs in winter were also much higher than those in summer. Unlike NPs, which are largely affected by temperature, it has been reported that the proportion of NCs in the particulate phase was higher than 90%, whereas the proportion of NCs in the gas phase was very small [17,34]. Hence, the temperature difference between winter and summer had little effect on the gas–particle distribution of NCs. The significant difference in NC concentrations between winter and summer may be due to the fact that biomass burning for heating in winter emitted large quantities of NCs and their precursors, which in turn produced a large amount of NCs when the concentrations of NO*x* were high. As shown in Figure 2, the higher concentrations of K^+^ in winter supported the impact of biomass burning on NCs. Regarding the differences between urban and suburban sites, the concentrations of NCs in summer were similar at the two sites, whereas the mean concentration of NCs in winter in MY was about 3 times that in CD. The concentrations of K^+^ in CD and MY in winter were comparable, so the higher NCs concentrations in MY may be related to the secondary formation. In addition to NCs, the concentrations of 4N-NaP in winter were 8–20 times those in summer, which may also be ascribed to intensive biomass burning or coal combustion in winter. 4N-NaP is mainly derived from the secondary formation of precursors emitted from coal combustion and biomass burning under high NO*x* conditions. The higher K^+^ and Cl^−^ concentrations in winter in Figure 2 confirmed that these two sources contributed significantly to 4N-NaP, since K^+^ is typically emitted from biomass burning, and Cl^−^ usually originates from coal combustion [35,36,37]. Similarly, the concentration of 4N-NaP in MY in winter was about twice that of CD, but they were similar in summer. The concentrations of NPs and NSAs in MY were about 1.1 and 1.2 times higher than those in CD, respectively, while they were comparable in summer at both sites.

In this study, only NPs in the particulate phase were determined. Considering the high abundance of NPs in the gas phase, the effect of temperature on NP levels in PM_2.5_ may be more significant than the influence of emission sources. Both CD and MY are located in the Sichuan Basin, where meteorological conditions are similar, resulting in similar NPs levels in PM_2.5_ at the two sampling sites. Unlike NCs and 4N-NaP, which were greatly influenced by emission sources and secondary formation, the higher concentrations of NPs in winter than in summer may be related to the significant difference in winter and summer temperatures. Lower temperatures in winter favored the distribution of NPs in the particulate phase; conversely, high temperatures in summer allowed more NPs to be present in the gas phase, resulting in lower concentrations in the particulate phase. Among the four categories, NSAs showed the least variation between seasons and sites. Photochemical oxidation of volatile organic compounds emitted from anthropogenic sources was the primary source of NSAs. Concentrations of NSAs in CD and MY were higher in winter than in summer, which may be mainly influenced by unfavorable meteorological conditions in winter, such as low wind speed and low boundary layer, which led to the accumulation of pollutants and higher concentrations in winter.

### 3.2. Light Absorption Properties of NACs

**Figure 6:**
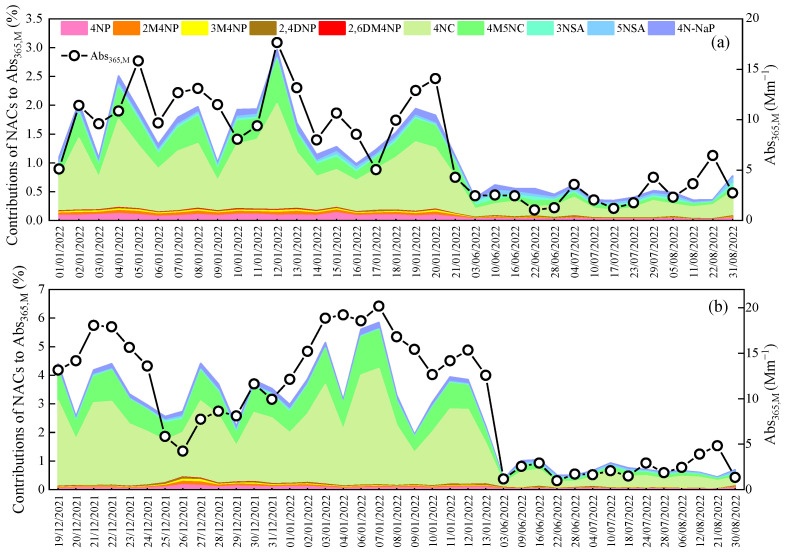
Time series of Abs_365,M_ of methanol extracts and the contributions of 10 NACs to Abs_365,M_ in CD (**a**) and MY (**b**).

Methanol solvent was used to extract NACs from PM_2.5_ filters. Hence, the methanol extracts were used as a substitute for atmospheric BrC, and the contributions of 10 NACs to the light absorption of methanol extracts were estimated in this study. In order to avoid nitrate interference, the absorption coefficient at 365 nm is typically used to evaluate the light absorption of atmospheric BrC. As shown in Figure 6, in CD, the light absorption coefficient of BrC at 365 nm (Abs_365,M_) ranged from 4.3 to 17.6 Mm^−1^ in winter and 1.0 to 6.4 Mm^−1^ in summer, with mean values of 10.5 ± 3.4 Mm^−1^ and 2.7 ± 1.4 Mm^−1^, respectively. In MY, Abs_365,M_ varied from 4.2 to 20.2 Mm^−1^ and 1.0 to 4.8 Mm^−1^ in winter and summer, with mean values of 13.6 ± 4.3 Mm^−1^ and 2.3 ± 1.1 Mm^−1^, respectively. In terms of seasonal variations, the Abs_365,M_ values of methanol extracts in winter were about 4–7 times higher than those in summer, suggesting more light-absorbing compounds being present in winter, or the organic matter being more capable of absorbing light in winter. Regarding the different sampling sites, Abs_365,M_ in MY was higher in winter but slightly lower in summer than in CD, suggesting that the light-absorbing properties of BrC depended on both sampling locations and seasons.

**Figure 7:**
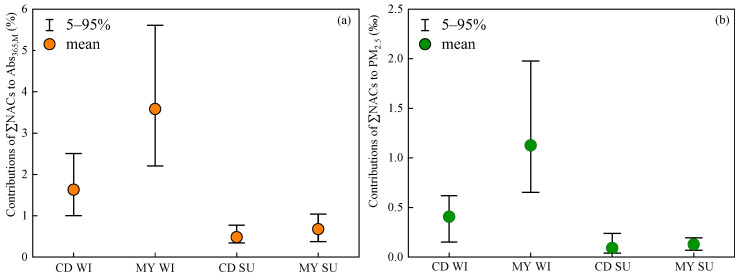
Contributions of ΣNACs to Abs_365,M_ (**a**) and PM_2.5_ mass concentrations (**b**) in winter (WI) and summer (SU) in CD and MY.

In order to evaluate contributions of NACs to the light absorption of BrC, the MAE_365_ value of 2,4DNP determined by Zhang et al. [38], and the remaining nine NAC MAE_365_ values from Yuan et al. [39] were used to calculate the light absorption coefficient of each NAC according to Equation (2). As shown in Figure 6 and Figure 7, in winter, contributions of ΣNACs to the light absorption of BrC fluctuated greatly during the sampling period in 2022, where daily contributions ranged from 1.0% to 3.0% with an average contribution of 1.6% in CD and from 1.9% to 5.8% with a mean contribution of 3.6% in MY. In summer, daily contributions of ΣNACs to Abs_365,M_ varied from 0.3% to 0.8% in CD and 0.4% to 1.1% in MY, averaging 0.5% and 0.7%, respectively. Although the values of Abs_365,M_ were higher in winter than in summer, the contributions of ΣNACs to the Abs_365,M_ of atmospheric BrC were also higher in winter, suggesting that the strongly light-absorbing NACs were more predominant in winter. Considering the site differences, contributions of ΣNACs to Abs_365,M_ in MY were higher than those in CD in both winter and summer, again suggesting that NACs in the two cities may have different sources or formation mechanisms.

Compared with data collected by Huang et al. [24] in six megacities in 2019–2021, contributions of ΣNACs to Abs_365,M_ in winter in this study were lower than those in Xi’an (7.98%), Guangzhou (7.89%), Harbin (7.11%), and Wuhan (4.18%) and comparable to Chengdu (1.74%), but higher than that in Beijing (0.85%), while contributions of ΣNACs to Abs_365,M_ in summer in this study were much lower than in those megacities, such as Xi’an (2.66%), Guangzhou (6.44%), Harbin (2.51%), Wuhan (3.00%), Chengdu (2.26%), and Beijing (2.07%). The distinct difference in contributions of ΣNACs to Abs_365,M_ between our study and Huang et al. [24] could be caused by different NAC levels in different regions and the MAE_NACs,365_ values used for calculating the light absorption coefficient of individual NACs. The MAE_NACs,365_ values for some NACs obtained by Huang et al. [24] through Gaussian software were higher than the MAE_NACs,365_ obtained from Yuan et al. [39], especially for 4NC and 4M5NC, which may partly explain the higher contributions of ΣNACs to the total light absorption of BrC than those in this study. Furthermore, contributions of ΣNACs to Abs_365,M_ in this study were higher than those in Xi’an in winter (0.82%) and summer (0.09%) in 2015–2016 [39], and comparable with that in Nanjing [23]. Due to the lack of data on the mass concentrations of OC, the contribution of ΣNACs to PM_2.5_ mass was calculated. As can be seen in Figure 7, the mean contributions of ΣNACs to PM_2.5_ mass concentrations in CD and MY were 0.4‰ and 1.1‰ in winter and about 0.1‰ in summer, respectively. Contributions of ΣNACs to Abs_365,M_ were about 35–80 times their contributions to PM_2.5_ mass concentrations, indicating that NACs were important light-absorbing components of atmospheric BrC in Sichuan Basin.

**Figure 8:**
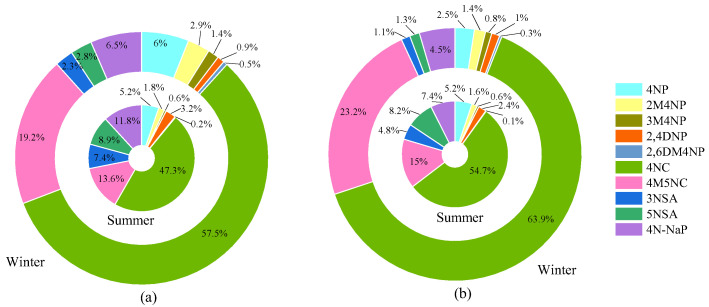
Contributions of 10 NACs to the light absorption of ΣNACs in winter (external) and summer (internal) in CD (**a**) and MY (**b**).

Among the 10 NACs, 4NC contributed the most to the light absorption of ΣNACs. As shown in Figure 8, the contribution of 4NC to the total light absorption of 10 individual NACs (Abs_ΣNACs,365_) was up to 63.9% in winter and 54.7% in summer in MY, as well as 57.5% and 47.3% in CD, respectively. For other NACs, their contributions to Abs_ΣNACs,365_ were less than 25%. For example, 4M5NC accounted for 13.6–23.2% of Abs_ΣNACs,365_ during the sampling period; the percentages of NPs in Abs_ΣNACs,365_ were less than 6% in both winter and summer at the two sampling sites. For NSAs, the sum of 3NSA and 5NSA accounted for about 15% of Abs_ΣNACs,365_ in summer, but less than 6% in winter; the contribution of 4N-NaP to Abs_ΣNACs,365_ was higher in summer (7.4–11.8%) than in winter (4.5–6.5%). Overall, NCs are important light-absorbing substances for atmospheric BrC in Sichuan Basin. There are two reasons to explain the higher contribution of NCs to Abs_ΣNACs,365_: firstly, the MAE_NACs,365_ of 4NC and 4M5NC was higher, by about 2–4 times that of NSAs and NPs [39]; secondly, the concentrations of 4NC and 4M5NC were higher than those of other NACs as well. As shown in Table 1, the concentration of 4NC in MY was about 10–40 times higher than those of other NACs. Regarding the mass fractions of NACs to PM_2.5_, except for 4NC, 4M5NC, and 4N-NaP, the percentage of the other NACs in Abs_ΣNACs,365_ was lower than that in the mass concentration, especially for 4NP, in which the contribution of 4NP to Abs_ΣNACs,365_ was about 3-fold lower than its contribution to the mass concentration.

### 3.3. Potential Source Regions of NACs

**Figure 9:**
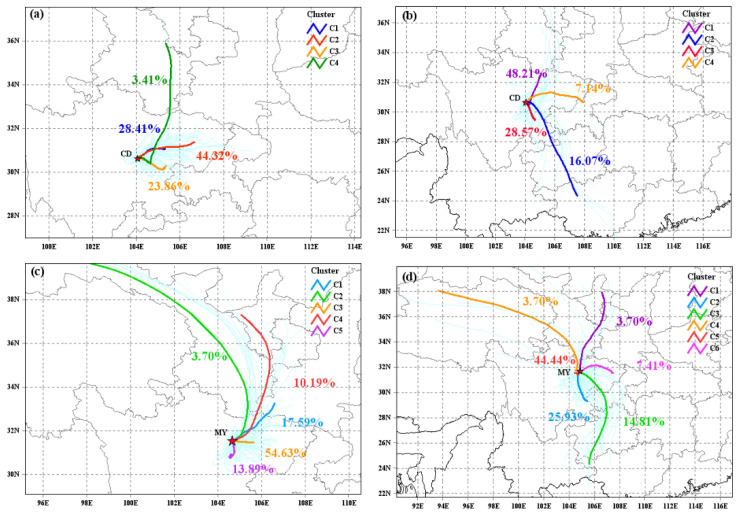
48-h airmass backward trajectories and clustering analysis in winter (**a**,**c**) and summer (**b**,**d**) in CD (**upper panel**) and MY (**lower panel**), respectively.

To further identify the geographical regions of NAC sources in CD and MY, 48-h air mass backward trajectories were determined during the sampling period, and the results for trajectory clusters are shown in Figure 9. Considering the significant contributions of NCs to the light absorption of BrC, Table 2 summarizes the mean concentrations of 4NC, 4M5NC, and ΣNACs associated with each cluster. In CD, four clusters were identified in both winter and summer, while in MY, five clusters were found in winter, and six clusters were observed in summer. As shown in Figure 9, air trajectories in winter at both sampling sites mainly originated from within the Sichuan Basin, which was mainly ascribed to the topography of the basin and the stable winter weather. In winter, air trajectories in CD mainly originated from the northeast of the sampling site (cluster C1 and C2), together accounting for about 73% of the total number of trajectories. The concentrations of ΣNACs, 4NC, and 4M5NC associated with cluster C1 were also the highest, while the NAC concentrations associated with cluster C3 from the southeast were the lowest. Although the proportion of air trajectories in cluster C4 was lower, the mean concentration of ΣNACs carried by these trajectories was only slightly lower than that of cluster C1, indicating that pollutants emitted from residential heating in northwestern China may be transported long distances to Chengdu. In summer, the predominant trajectories in CD were from the northeast, with cluster C1 accounting for about 48.2%; followed by the southeast, with clusters C2 and C3 together accounting for about 44%; and cluster C4 from the eastern part of CD accounted for only 7%. As shown in Table 2, the mean concentrations of ΣNACs carried by cluster C2 and C3 were higher, as was the case for 4NC and 4M5NC, suggesting that the main sources of NACs were distributed in the southeast of the sampling site in summer.

The clustering results for the air mass backward trajectories in MY were slightly different from those in CD. As shown in Figure 9c, cluster C3, which was from local and nearby areas of MY, accounted for the highest proportion of the total number of trajectories during the observation period. The trajectories of cluster C2 from the north of MY showed the lowest contribution (only 3.7%), and the remaining three air mass trajectories accounted for 10–18%. Regarding the variations in ΣNAC concentrations associated with each cluster, the mean concentration of ΣNACs from cluster C3 was the highest, with the sources of NACs mainly distributed east of the sampling site. Furthermore, the mean concentration of ΣNACs associated with cluster C5 was also high, indicating that local emissions contributed the most to NACs in MY. Although the proportion of cluster C2 was relatively low, the mean concentration of ΣNACs carried by these trajectories was still higher than the other three clusters. This was similar to the long-range transport of air trajectories in CD, again demonstrating that pollutants from northwestern China were transported to the Sichuan Basin over long distances. In summer, the air trajectories primarily originated from the local MY, of which cluster C5 accounted for about 44%, followed by the south (C2) and southeast (C3) of the sampling site, which together accounted for about 40% of the total number of trajectories. However, air trajectories from the other three directions contributed less than 10% each. As shown in Table 2, the trajectories in cluster C2 carried the highest concentrations of ΣNACs and NCs, followed by cluster C5, which was related to local emissions. The other four clusters carried lower concentrations of NACs, suggesting that potential source regions of NACs in MY were distributed around the sampling site.

## 4. Conclusions

Ten NACs (such as nitrophenols, nitrocatechols, nitrosalicylic acids, and nitronaphthol) in PM_2.5_ samples collected in winter and summer of 2022 in the urban (CD) and suburban (MY) areas of the Sichuan Basin were analyzed. The seasonal mean concentration of ΣNACs in winter at the suburban site was 2.4 times higher than that in urban CD, whereas ΣNAC concentrations in summer were similar at the two sites. Among the 10 NACs, 4NC was the most abundant, accounting for 44.3% and 35.6% of ΣNAC concentration in CD in winter and summer, respectively, and 56.2% and 42.1% in MY. Through clustering analysis of the air mass backward trajectories, the source regions of NACs were mainly distributed in the Sichuan Basin, although NAC levels were occasionally affected by the long-range transport of pollutants from northwestern China.

The Abs_365,M_ values for atmospheric BrC in winter were about 4–7 times those in summer, indicating that organic matter contained more light-absorbing substances or that the organic matter in winter had stronger light-absorbing capacity. Regarding different sampling sites, the value for Abs_365,M_ in MY in winter was higher than that in CD, while it was slightly lower than that in CD in summer. In winter, the mean contribution of ΣNACs to the Abs_365,M_ of BrC was 1.6% in CD and 3.6% in MY, respectively; in summer, they were 0.5% and 0.7%, respectively. The contributions of ΣNACs to the light absorption of BrC were about 35–80 times those of the mass fractions in PM_2.5_, implying that nitroaromatic compounds were important light-absorbing components of atmospheric BrC in Sichuan Basin. Among the 10 NACs, 4NC contributed the most to the light absorption of ΣNACs, followed by 4M5NC and 4N-NaP. Therefore, controlling the emissions of NCs and 4N-NaP from biomass burning and coal combustion and/or the precursors that form these NACs can effectively reduce the impact of atmospheric BrC in Sichuan Basin on climate change.

## Figures and Tables

**Table 1:** Concentrations of NACs in CD and MY in winter and summer of 2022.

		Concentrations (ng m^−3^)										
		4NP	2M4NP	3M4NP	2,4DNP	2,6DM4NP	4NC	4M5NC	3NSA	5NSA	4N-NaP	ΣNACs
CD winter	Max	7.1	3.2	1.8	0.06	0.6	41.0	18.1	1.4	1.8	2.4	77.0
	Min	1.1	0.4	0.3	0.01	0.06	3.4	1.2	0.3	0.4	0.3	7.7
	Mean	4.5 ± 1.6	1.7 ± 0.8	0.9 ± 0.5	0.04 ± 0.01	0.3 ± 0.2	13.6 ± 8.8	5.2 ± 3.9	0.9 ± 0.3	1.3 ± 0.4	1.2 ± 0.6	29.5 ± 16.2
CD summer	Max	0.6	0.2	0.05	0.02	0.04	2.0	0.4	0.6	0.7	0.2	3.7
	Min	0.1	0.03	0.01	0.00	0.00	0.2	0.1	0.05	0.1	0.05	0.7
	Mean	0.3 ± 0.1	0.08 ± 0.04	0.03 ± 0.01	0.01 ± 0.01	0.01 ± 0.01	0.8 ± 0.6	0.2 ± 0.08	0.2 ± 0.1	0.3 ± 0.2	0.1 ± 0.07	2.2 ± 1.1
MY winter	Max	7.6	3.4	2.4	0.2	0.5	103.8	36.9	1.8	2.6	4.6	159.7
	Min	2.4	1.0	0.6	0.07	0.1	8.4	2.6	0.7	0.8	0.7	19.7
	Mean	4.6 ± 1.2	2.0 ± 0.6	1.2 ± 0.4	0.1 ± 0.04	0.3 ± 0.1	42.1 ± 24.4	16.5 ± 9.0	1.2 ± 0.3	1.5 ± 0.4	2.2 ± 1.0	71.7 ± 35.6
MY summer	Max	0.5	0.2	0.04	0.04	0.01	2.2	0.7	0.4	0.8	0.2	4.9
	Min	0.1	0.03	0.01	0.00	0.00	0.2	0.08	0.06	0.1	0.05	0.8
	Mean	0.3 ± 0.1	0.08 ± 0.03	0.03 ± 0.01	0.01 ± 0.01	0.01 ± 0.01	1.1 ± 0.7	0.3 ± 0.2	0.2 ± 0.09	0.3 ± 0.2	0.1 ± 0.05	2.5 ± 1.2

**Table 2:** Mean concentrations of NCs and ΣNACs carried by different air mass trajectories in CD and MY.

		Mean Concentrations (ng m^−3^)					
		C1	C2	C3	C4	C5	C6
CD winter	4NC	17.6	12.6	10.3	15.6		
	4M5NC	6.8	4.9	3.6	5.2		
	ΣNACs	36.2	28.2	23.2	33.9		
CD summer	4NC	0.6	1.2	1.3	0.2		
	4M5NC	0.2	0.2	0.3	0.1		
	ΣNACs	1.8	2.4	3.1	1.0		
MY winter	4NC	30.6	43.7	51.3	20.4	36.6	
	4M5NC	12.7	14.9	20.4	7.6	13.4	
	ΣNACs	54.9	72.6	85.5	37.9	64.5	
MY summer	4NC	0.7	1.5	0.7	0.6	1.0	0.3
	4M5NC	0.3	0.3	0.2	0.2	0.3	0.1
	ΣNACs	1.9	3.2	1.7	1.8	2.4	1.0

## Data Availability

Data are contained within the article.
